# Smartphone dependence and its influence on physical and mental health

**DOI:** 10.3389/fpsyt.2025.1281841

**Published:** 2025-08-19

**Authors:** Wei Zhu, Ying Zhang, Yanzhi Lan, Xinqiang Song

**Affiliations:** ^1^ School of Medicine, Xinyang Normal University, Xinyang, China; ^2^ Xinyang Central Hospital, Xinyang, China

**Keywords:** smartphone dependence, dopamine signaling, sleep, depression, self-control, exercise, mobile phone, cell phone

## Abstract

Smartphones have become an integral part of life for an increasing number of people around the world, especially as the range and speed of smartphone functions has expanded. However, excessive use of smartphones can cause not only physical discomfort but also feelings of loneliness, anxiety, and depression. The present review explores the negative effects of excessive smartphone use on physical and mental health. It also surveys measures that can counteract these effects, which include controlling smartphone use, strengthening self-control, and engaging in physical exercise.

## Highlights

- Smartphones have become an integral part of modern life.
- Excessive use of smartphones can cause unhealthy physically and mentally.
- Feeling of discomfort include physical discomfort and feelings of loneliness, anxiety, and depression.
- Measures include controlling smartphone use, strengthening self-control, and engaging in physical exercise.

## 1 Introduction

Smartphones have become an integral part of modern life (1, 2), offering much richer possibilities for communication than the voice calling offered by earlier generations of telephones or mobile phones. Smartphones enable video calls and Internet access for listening to music, watching videos, shopping and accessing information—all in a format more portable and flexible than a desktop computer or even laptop (3–7). In China alone, the number of mobile Internet users was estimated at just over 1 billion in December 2022, which was 36 million more than in December of the previous year, and more than 99% of people in China report accessing the Internet *via* smartphone (8, 9). The range and portability of Internet services offered by smartphones have made them essential for people who wish to keep pace with the rapid developments of human society and the Internet. Updates and upgrades to smartphones have become a way to express one’s individuality and style.

**Figure 1:**
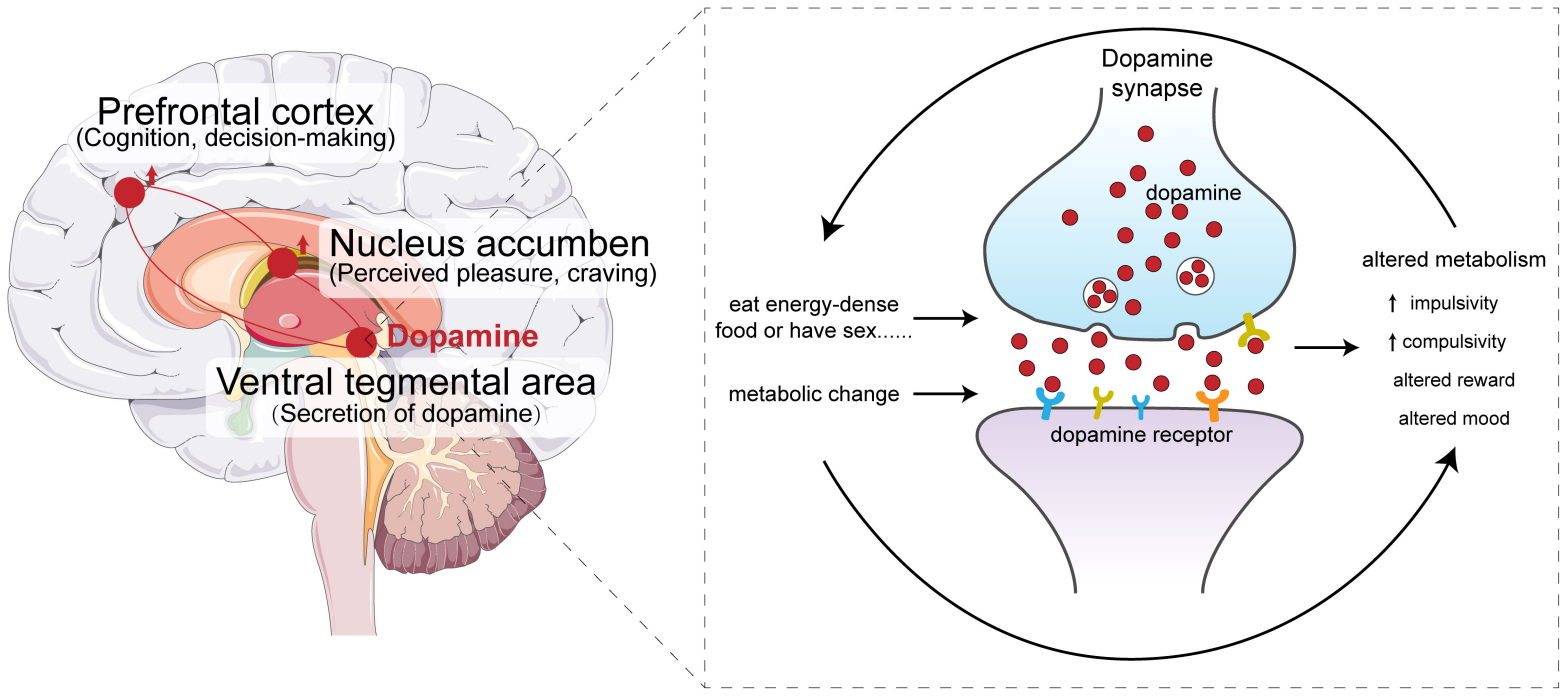
The hypothesis of smartphone addiction.

Precisely because of their multifunctionality, smartphones have created a new form of dependence: whether waiting for a bus or subway, eating a meal, watching TV or preparing for bed, an increasing number of people rely on their smartphones to help them pass the time (10, 11). The growing dependence on smartphones has led to the so-called “heads-down” generation, whose physical and mental well-being may be harmed in several ways (12, 13). Excessive smartphone use, also referred to as “mobile phone dependency”, “mobile phone addiction”, or “problematic mobile phone use” can lead to various psychological, physiological, and social impairments (14–17) (**Figure 1**).

It is important to note that addiction, including smartphone addiction, is a result of the interaction between genetics and environment. Even if genetic factors increase susceptibility (e.g., making some people more sensitive to the reward of smartphone use or less able to control impulses), environmental factors (e.g., family upbringing, social context, and daily usage habits) play a more direct role in triggering and maintaining the behavior. At present, there is no sufficient evidence to confirm that a single or specific set of genes determines the risk of smartphone addiction.

From a theoretical perspective, smartphone addiction, as a type of non-substance addiction, shares similarities with substance addiction in terms of behavioral characteristics (e.g., compulsive use, reward-seeking) and potential neurobiological mechanisms (e.g., involvement of the brain’s reward circuit and monoamine neurotransmitter systems). Since substance addiction is known to have a genetic component (e.g., genes related to dopamine receptors like DRD4, DRD2, or serotonin transporters like 5-HTTLPR (5-hydroxytryptamine transporter linked polymorphic region, 5-HTTLPR) have been linked to addiction susceptibility) (18–20), researchers speculate that similar genetic factors may also influence the risk of smartphone addiction.This review discusses the various negative impacts on well-being that have been reported for excessive smartphone use, and it explores interventions and measures to mitigate or prevent those impacts.

## 2 Methods

We carried out a literature search in Pubmed/MEDLINE, Web of Sciences, Scielo and LILACS search using the following MeSH entry terms: “smartphone”, “mobile phone”, “cell phone”, “physical health”, “mental health”, “depression”, “sleep”,”loneliness”, “anxiety” and “dopamine signaling”, “musculoskeletal”, “low vision”, “ophthalmological”and we generated this search strategy for Pubmed: ((“smartphone t”[MeSH Terms]) OR (“mobile phone “[MeSH Terms]) OR (“cell phone” [MeSH Terms])AND (“depression” [MeSH Terms]) OR(”sleep “[MeSH Terms]) OR (”loneliness “[MeSH Terms])OR(”anxiety “[MeSH Terms])OR(”dopamine signaling “[MeSH Terms]) OR (”anxiety “[MeSH Terms])OR(”musculoskeletal “[MeSH Terms])OR (”low vision “[MeSH Terms])OR(”ophthalmological “[MeSH Terms]) OR(”mental health “[MeSH Terms])OR(”physical health “[MeSH Terms])).

We used equivalent strategies in the other databases. We reviewed the articles published between 2002 and August 2023, and no limitation for any language was used. The reference lists of the selected articles were also evaluated to identify other publications. The present study followed the PRISMA guidelines (19). As this study was not quantitative or epidemiological but a descriptive study, we did not assess the selected studies’ quality and did not perform a meta-analysis. An ethical analysis was not needed since it was a literature searching-based study.

## 3 Problems associated with excessive smartphone use

### 3.1 Addiction to dopamine signaling

**Figure 2:**
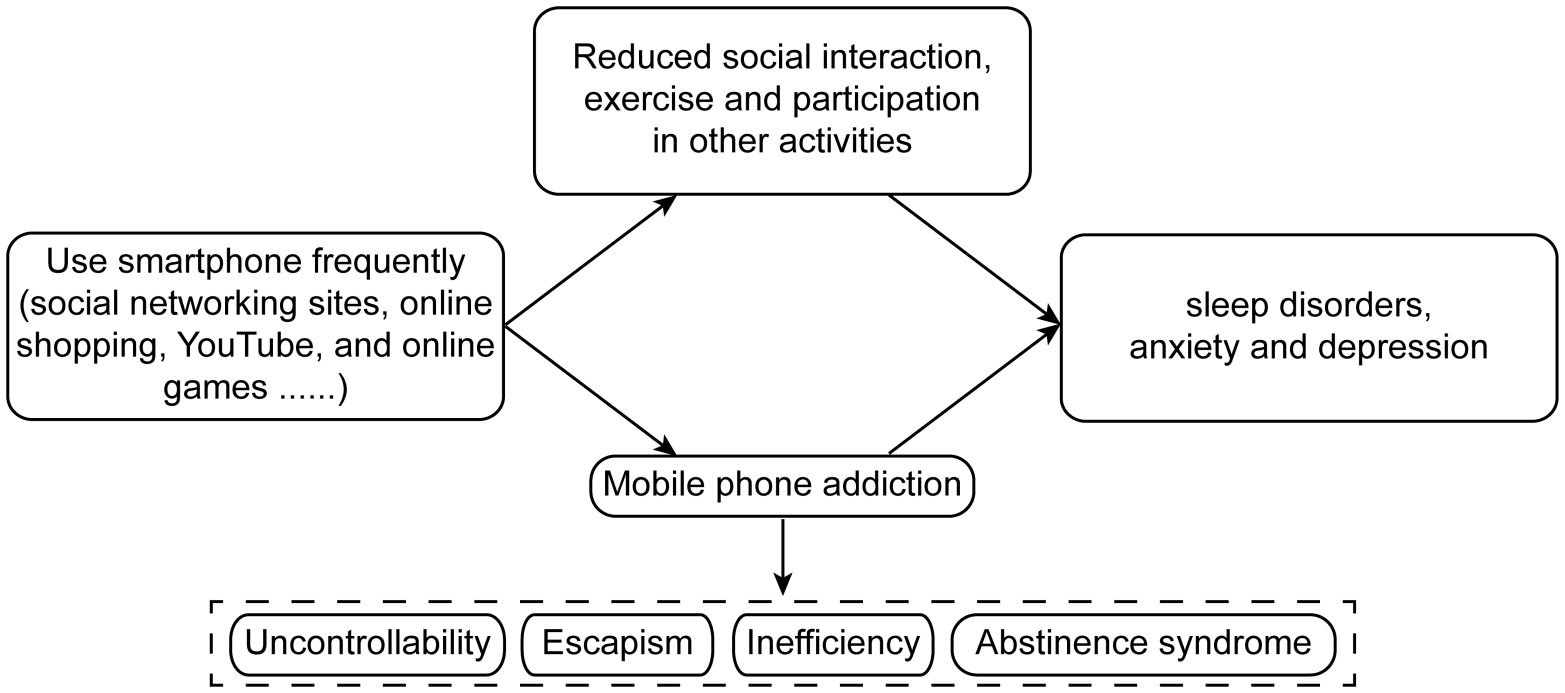
Possible pathways of addiction to dopamine signaling.

Dopamine plays a crucial role in the brain’s reward system, which drives us to take actions to ensure our survival and pass on our genes to future generations. When we eat or have sex, the brain rewards us with a pleasant release of dopamine, so we strive to repeat those activities in the future in order to experience the same dopamine “rush”. We also experience this rush when we explore unfamiliar places, make new friends, or try things we have never done before, which may have evolved to drive us to explore in order to secure food and safety (21, 22) (**Figure 2**). We experience this rush when we learn something new, probably reflecting the evolutionary truth that the more we know about the world, the greater our chances of survival. For example, knowing how weather affects lion movements or when gazelles let down their guard can improve our hunting success (23). When confronted with something new, the brain releases dopamine to enhance focus and learning. There are numerous dopamine-producing cells in the brain that respond to novel stimuli but remain quiet in the face of familiar things (24, 25).

Today most of us no longer need to migrate in search of food, so the smartphone offers us an alternative route to the dopamine rush by providing us with a constant, easily accessible source of information (26) (27). Whenever our smartphone notifies us of a new message or development in the Internet, we cannot resist the urge to check it immediately. The reward circuit with the nucleus accumbens as the core plays a crucial role in smartphone addiction. When people obtain positive experiences from smartphones, the reward circuit is activated, and dopaminergic neurons in the nucleus accumbens release dopamine, interacting simultaneously with brain regions such as the prefrontal cortex and the amygdala. The prefrontal cortex is responsible for high-level cognitive functions such as decision-making and impulse control, while the amygdala is involved in emotional processing. In the case of smartphone addiction, the excessive activation of the reward circuit leads to a decrease in the prefrontal cortex’s ability to control impulses. At the same time, the activity of the amygdala increases, making individuals more prone to strong emotional responses and cravings for smartphone-related stimuli, and it becomes difficult for them to suppress the urge to use smartphones. Every time we open a new webpage, the brain releases dopamine, driving us to constantly open new pages without stopping; the “next page” always seems better than the one we just saw. We spend fewer than 4 seconds on each webpage, and only 4% of webpages hold our attention longer than 10 minutes (28).

Smartphone addiction and drug addiction have similar manifestations: both involve a strong craving for the target object (drugs/smartphones), leading to uncontrollable and repeated use, and both are related to the activation of the brain’s reward circuit (such as the dopamine system). However, they also have differences. Drug addiction is driven by chemical substances, with strong physical dependence—long-term use leads to obvious physical withdrawal symptoms (such as vomiting, tremors) after cessation, directly causing organic damage, requiring medical intervention for withdrawal. In contrast, smartphone dependence is driven by digital stimuli and psychological-behavioral mechanisms, with almost no physical dependence; withdrawal mainly causes psychological discomfort (such as restlessness) without severe physical reactions. Its health risks are mostly indirect (like vision loss, sleep disorders), and withdrawal can generally be improved through behavioral adjustments (29–31).

### 3.2 Poor sleep quality

While the precise function of sleep remains poorly understood, it clearly is important because otherwise it would have been far too dangerous for early humans to spend nearly one-third of the day in an unconscious state, when they were more susceptible to predators and could not search for food, reproduce or otherwise work toward survival. Sleep is not important for helping us save energy: the brain (32) consumes a similar amount of energy during sleep as during wakefulness. Instead, one key function of sleep appears to be allowing the brain to clear protein waste that has accumulated during the day. In fact, the weight of metabolic waste cleared by the brain in a year may be equivalent to one’s body weight (33). Such lack of cleaning may help explain why chronic sleep deprivation can increase the risk of stroke and dementia (34). Another key function of sleep appears to be the “consolidation” of short-term memories into long-term memory (35): during sleep, the brain selects certain events of the day to store as long-term memories, while it also retrieves lost memories.

In China, one of every two people sleeps fewer than the recommended 7–9 hours per night, and the situation appears to be similarly bad in other countries (36). Lack of sleep affects our performance: sleeping fewer than 6 hours on 10 consecutive days decreases our attention to a similar extent as not sleeping for 24 hours (37). Sleep deprivation also intensifies and destabilizes our emotional responses in the amygdala, which drives our responses to stress (38).

By stimulating us even into late hours of the night, smartphones can rob us of the sleep that we need. The brain remains awake under constant stimulation by apps, social media, and other sources of information that trigger dopamine secretion (39). A study of more than 600 adults linked more time on electronic devices such as smartphones to worse sleep quality (40). Participants who reported staying up late to use their smartphones reported difficulties falling asleep and continuously declining sleep quality, leading to fatigue the next day.

The light of the smartphone screen can trick our body into thinking that it is daytime, inhibiting production of the melatonin that tells our body to sleep (41) (42). In fact, the blue light from smartphone screens inhibits melatonin production much more than the white light from ambient lighting (43, 44). As a result, the use of smartphones just before bed delays one’s falling asleep by 2–3 hours, shifting one’s entire circadian rhythm accordingly.

However, smartphones can compromise our sleep even when we are not watching the screen. In a survey of thousands of middle school students, those who reported sleeping next to their smartphones slept an average of 21 minutes less per night than those who did not (45). Based on that study, a smartphone in the same room disturbs sleep more than a television in the same room.

In addition, excessive use of mobile phones can cause stress and anxiety, which in turn reduce the duration and quality of our sleep (46).

Therefore, we should learn to suppress our craving to use smartphones, particularly in the evening before going to bed.

### 3.3 Depression

Large studies of adolescents in the US over the last decade have consistently linked greater use of digital products such as smartphones to greater risk of depressive emotions (47, 48). For example, adolescents who spend 6–9 hours per week with such products are significantly more likely to feel unhappy than those who spend 4–5 hours per week. The risk and severity of low mood increase with time spent on social networking sites, online shopping, YouTube, and computer games. Conversely, social interaction, exercise, playing musical instruments, and engaging in other activities have been linked to better mood (49, 50). That excessive smartphone use can cause sleep disturbances and depression was confirmed in a longitudinal study of 4,000 adolescents in US, which found that more frequent smartphone use led to higher levels of stress and rates of sleep disorders and depression (51, 52).

An analysis of over 125,000 children and adolescents in US showed that spending more than 2 hours a day on screens increases the risk of depression, and the risk increases with the duration of exposure (53, 54). Similar results were observed in a survey of 130,000 children and adolescents in China (55). A study of 40,000 children and adolescents in Europe found that those who used digital products longer than 7 hours a day were twice as likely to suffer depression or anxiety than those who spent less time in front of screens (56). The threshold of 7 hours per day is impressive because most adolescents have only 8–9 hours of leisure per day when they are not sleeping, studying or eating. It seems that nearly one of five adolescents spends nearly all their waking hours on digital devices (57). While reducing screen time can improve mood, it may be unrealistic to expect adolescents to limit themselves to the recommended one hour per day (58, 59).

**Figure 3:**
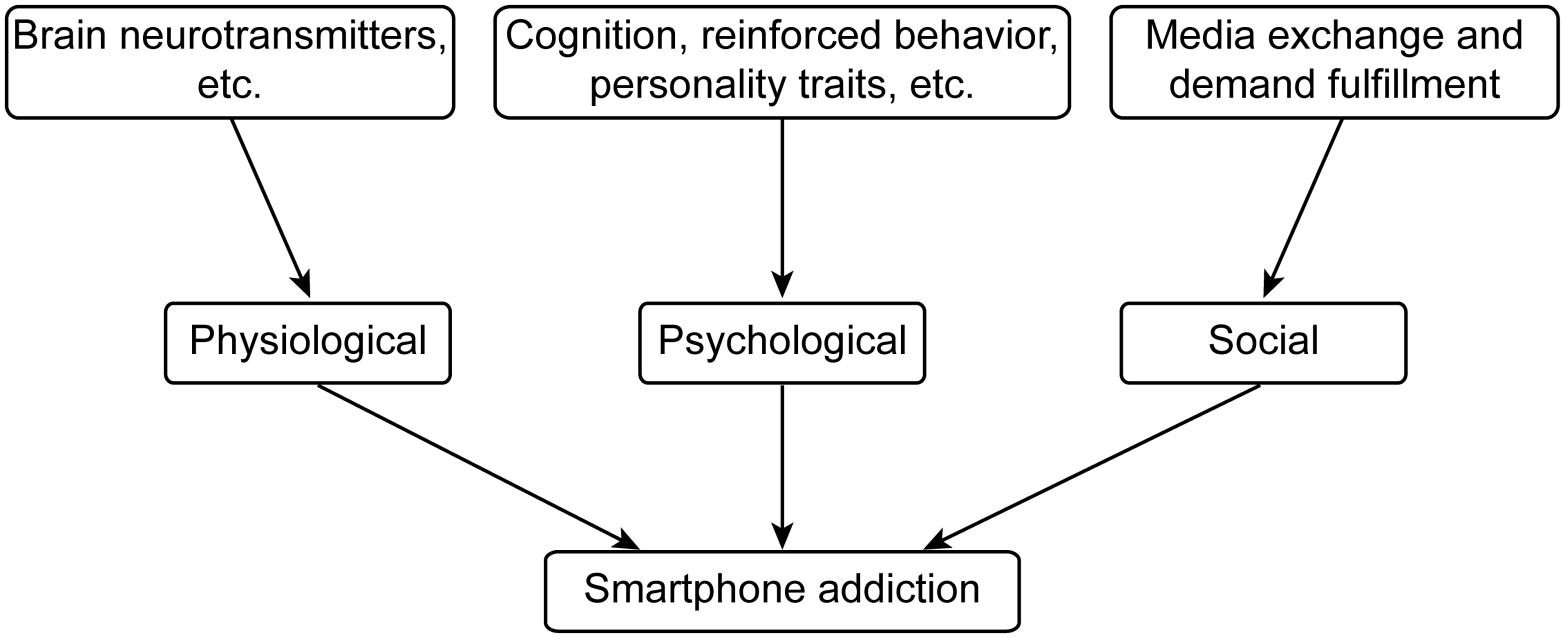
The association between mobile phone addiction and depression.

It is perhaps no coincidence that adolescents in the US have been reporting progressively stronger feelings of loneliness, worse sleep quality, and less socializing since 2011, when the Apple iPhone dominated the smartphone market with sales of 120 million units, which exceeded the sales for the preceding four years combined (60). At the same time, the documented increases in stress, depression and anxiety among adolescents in various countries should not be attributed entirely to smartphone use (**Figure 3**). Other factors, for example, likely include increased competitiveness to enter the job market and lower perceived job security (61).

Smartphone dependence and depression often show a bidirectional relationship rather than a one-way cause-effect link, though both directions of influence are supported by research (62–65).On one hand, smartphone dependence may contribute to depression. Excessive smartphone use can reduce in-person social interactions, leading to feelings of isolation; constant exposure to curated “perfect lives” online may trigger negative self-comparisons; disrupted sleep (due to late-night phone use) can also exacerbate emotional instability—all of which may increase the risk of depression. On the other hand, depression may also lead to smartphone dependence. People with depression may turn to smartphones as an escape: using social media, games, or videos to distract themselves from low mood, loneliness, or negative thoughts. Over time, this can develop into a reliance on phones as a coping mechanism. In short, they often reinforce each other: dependence may worsen depressive symptoms, and depression may deepen dependence (66).

### 3.4 Physical problems

Extended smartphone use has been shown to adversely affect musculoskeletal health, particularly in the neck and upper extremities (67). Young et al. (68) identified that device characteristics such as larger screen sizes and non-ergonomic grip configurations correlate with increased neck flexion angles and sustained wrist extension. Given the variations in device dimensions, weight distribution, and interaction patterns across different smartphones, these factors may lead to distinct symptom profiles among users. Empirical evidence further indicates that musculoskeletal discomfort most commonly manifests within the first 15–30 minutes of continuous smartphone use. Strategic behavioral modifications, such as adhering to 15-minute usage intervals followed by postural resets, could potentially prevent symptom onset in over 70% of habitual users. A study conducted in Zhuhai City, China, explored the relationship between smartphone usage behavior and low vision. Four primary schools were randomly chosen from four different districts of Zhuhai. A total of 462 fifth - grade students were recruited as survey participants. Employing a retrospective cohort study design, the research combined questionnaire surveys with physical examinations to investigate the associated factors of low vision among pupils. Logistic regression analysis was then used to identify the influencing factors of low vision. The findings showed that the detection rate of low vision among these primary school students was 56.71%. The study also revealed a significant correlation between pupils’ smartphone usage and the incidence of low vision. Low vision was influenced by multiple factors, including heredity, gender, the age at which students started using smartphones, and the daily duration of smartphone use (69).

## 4 Measures and interventions to counteract the health effects of excessive smartphone use

### 4.1 Medical methods to address smartphone addiction

Some medications can help regulate neurotransmitters in the brain to reduce the craving and dependence on smartphones. For example, drugs that act on the dopamine system, such as bupropion, may be used to adjust the brain’s reward mechanism and relieve the symptoms of addiction (70, 71). However, the use of drugs needs to be strictly under the guidance of a doctor, as different people may have different reactions and side effects. Cognitive - behavioral therapy (CBT) is a commonly used method (72, 73). It helps patients recognize and change their negative thinking patterns and behaviors related to smartphone use, set goals to limit smartphone use, and learn to cope with stress and emotions in other ways. Motivational interviewing is also an effective approach, which stimulates the patient’s internal motivation to change and enhances their confidence and determination to overcome addiction. Some neurological techniques, such as transcranial magnetic stimulation (TMS) (74, 75), can be used to modulate the activity of specific brain regions related to addiction. By applying magnetic fields to the prefrontal cortex and other areas, it is possible to improve the brain’s executive function and impulse control ability, thereby helping to reduce smartphone addiction. However, this method requires professional medical equipment and trained medical staff to operate.

### 4.2 Controlling smartphone use

One approach to helping young people reduce smartphone use is to encourage them to have a clear goal in mind when using it: the person should pick up the phone knowing what task he or she wants to accomplish, complete the task, then put the phone aside. This attitude may help the individual control the time and frequency of smartphone use. Another approach is to view one’s smartphone dependence as a normal feeling. This can reduce feelings of self-blame and encourage the individual to engage in mindfulness or meditation to enhance their ability to control their attention (76).

Since excessive smartphone use often goes hand-in-hand with impulsivity or anxiety, parents should be careful to notice when their children may be exhibiting either condition, in which case they should supervise and, if necessary, limit the children’s use of smartphones. Parents should not simply regard *all* use of smartphones as detrimental to their children, but should investigate the reasons why they wish to use the devices and help them engage with the devices constructively (47).

Educational institutions must struggle with how to keep the classroom sufficiently interesting so that students are not lured to their smartphones. This may be particularly difficult when students are in primary or secondary school, or when courses are quite specialized or theory-laden as at university. In these instances, formal regulations may be needed that regulate smartphone use in the classroom. It may be helpful to separate students physically from their smartphones, such as by asking them to deposit them inside “phone pouches” in every classroom, because the phones themselves trigger craving to check for new information or messages. Outside the university classroom, student counselors, as the school administrators closest to students, can strongly influence students’ awareness of smartphone addiction and their willingness and effectiveness in managing it (23, 77, 78).

### 4.3 Enhancing self-control

Improving one’s self-control can be quite effective for reducing impulsive behavior such as excessive smartphone use (79). Smartphone addicts must first become aware of their impulsive behavior and recognize it quickly when it occurs, so that they can activate their self-control systems to regulate it. Self-control can be regarded as a finite resource that can be increased through meditation, slow breathing, adequate sleep, and regular exercise (80–88). Developing good habits or automated behaviors can reduce the strain on finite self-control resources, reducing the risk of impulsive behavior (89).

Enhancing the self-control of university students can bring benefits that extend beyond smartphone use, improving their self-management ability in various domains of university life, when they no longer receive constant management from school officials or family members. Even so, parents should continue to support their children in enhancing self-control at university, such as by helping them to identify their shortcomings and to correct impulsive or unproductive behaviors (90, 91).

**Figure 4:**
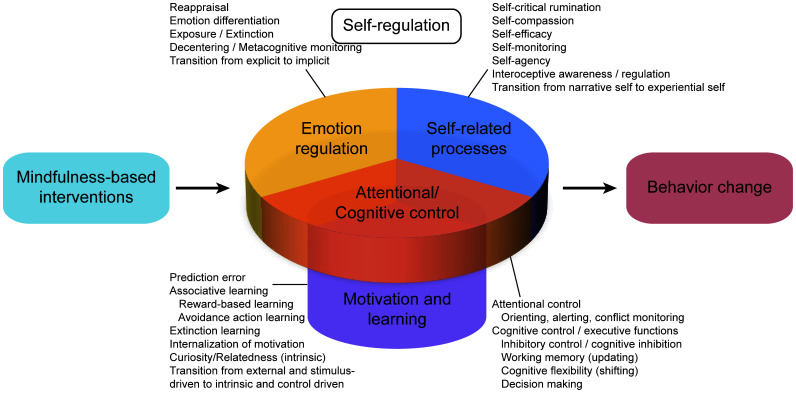
Mindfulness influences on self-regulation and behavior change.

The efforts of parents should be complemented by those of psychological health centers at universities (45), which can provide education aimed at increasing self-control and inhibiting impulsivity, routine screening for smartphone addiction, and monitoring of students based on their level of dependency with the help of a continuously updated database (92). Health centers should promote awareness of self-control and the need to enhance it through activities off-line and on-line *via* official social media accounts (**Figure 4**).

### 4.4 Engaging in exercise

A longitudinal study of 100 students 18–25 years old in Europe showed that after engaging in physical exercise for four weeks, most showed higher concentration, less lazy attitudes, and faster brain processing (93). A study of 3,000 adolescents in US showed that those who walked more during one week showed better attention. While these benefits of exercise can be detected even after occasional walks or jogging, consistent exercise for several weeks or months is necessary in order to effectively improve executive function (94, 95). One study found that university students in US could reduce their anxiety by walking 20 min per day, three times a week during two weeks, and the reduction persisted for up to one week after students stopped exercising (96). In that study, even stronger and equally persistent reduction was observed in a group of students who ran at high intensity for 20 min rather than walked during the two weeks. A review of nearly 100 studies judged to be rigorous concluded that walking, yoga, running, and muscle exercises can enhance cognition in adults xxx, particularly when the exercise is consistent (97). One study suggests that optimal effects can be achieved by engaging in at least 52 hours of exercise within six months, which breaks down to three 45-minute sessions per week. While low-intensity exercise such as leisure walking already brings benefits, exercise may be more effective when it increases heart rate (98).

**Figure 5:**
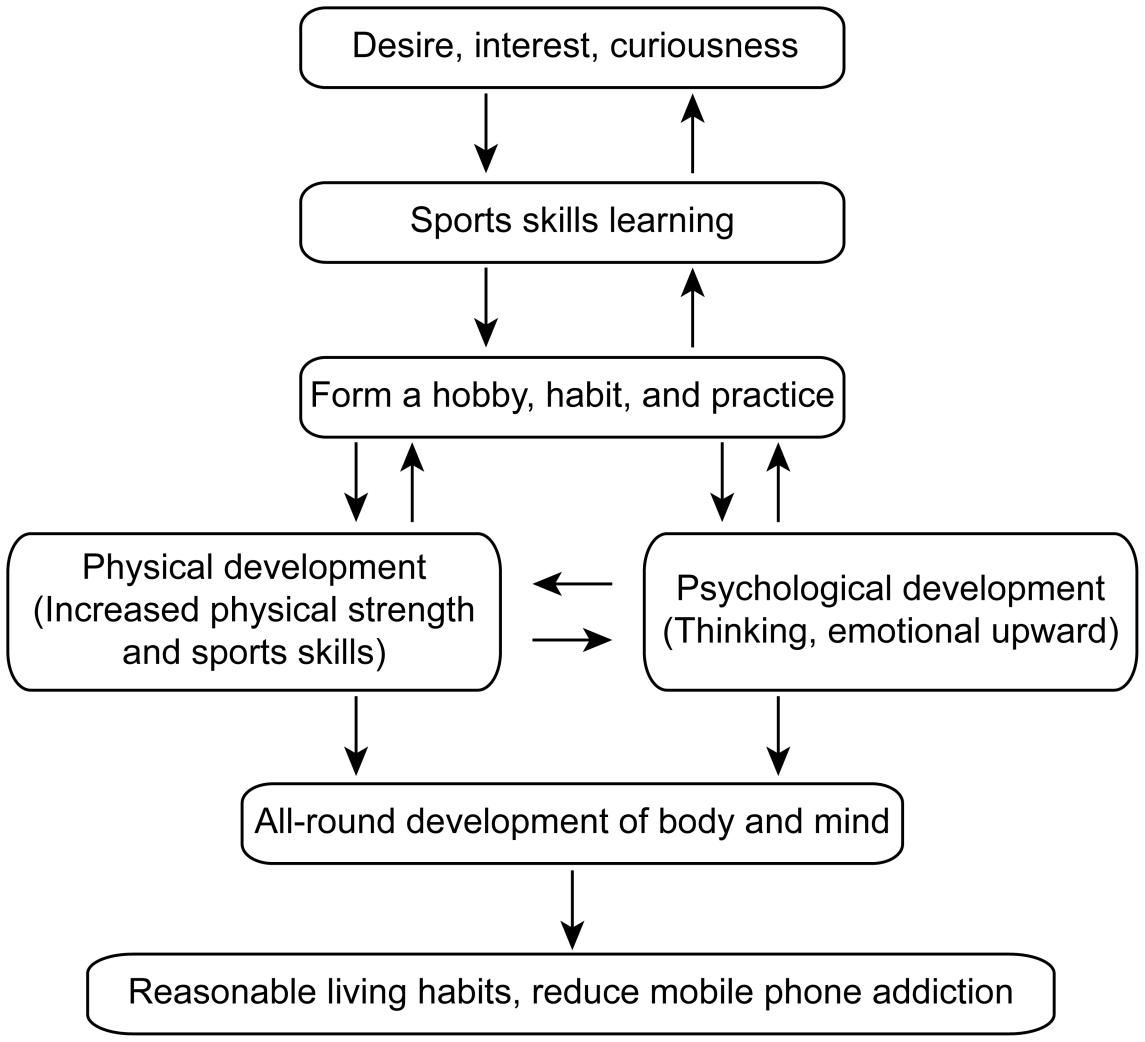
The relationship between sports skills and reducing mobile phone addiction.

**Figure 6:**
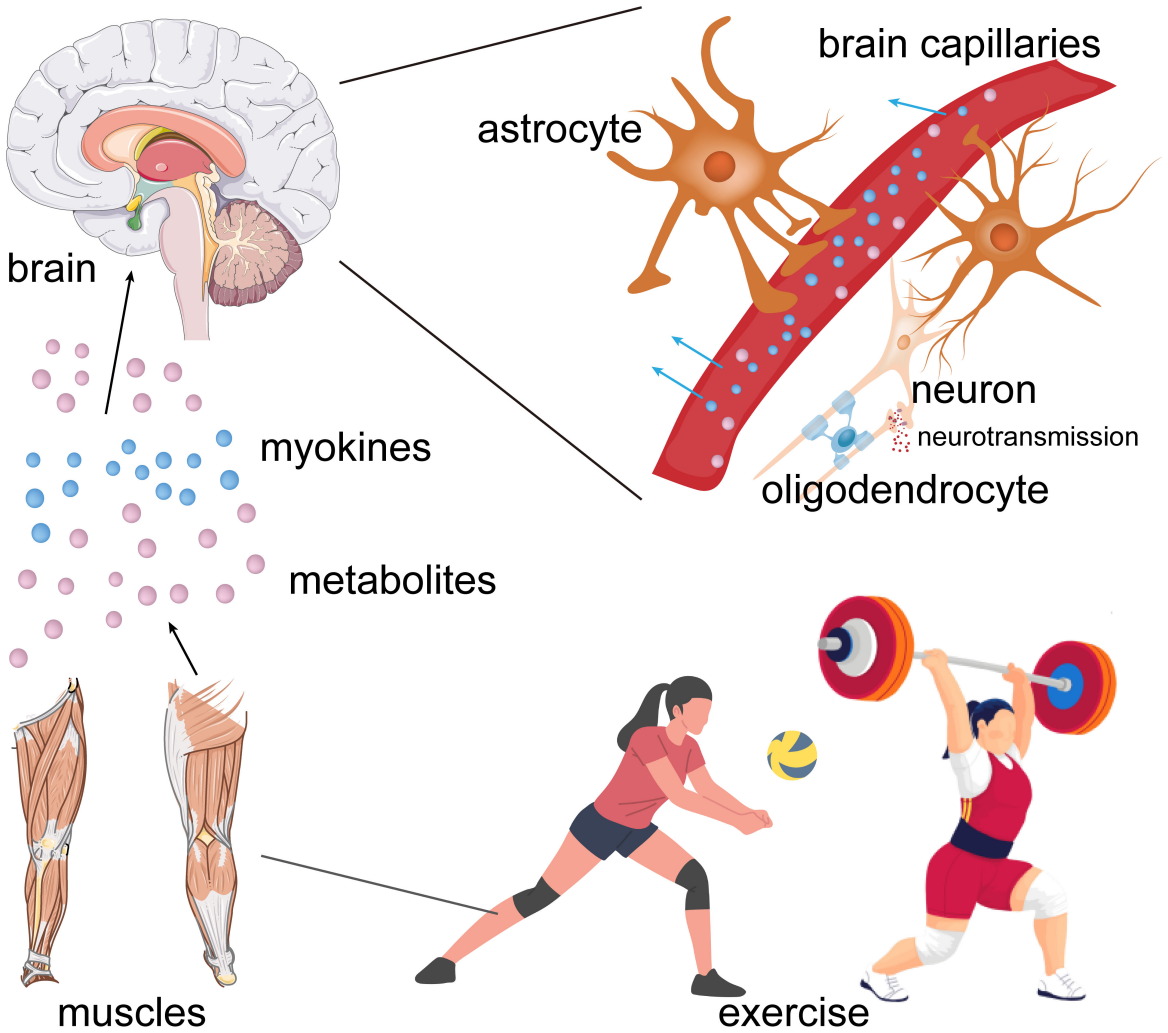
Possible pathway for the exercise-mediated effects on brain functions.

Why does exercise enhance focus? It may be because our ancestors needed to concentrate intensely during hunting or when avoiding predators, and these activities may easily occupied them for 2–3 hours per day (99). Since most of us do not require such sustained, intensive concentration for survival, we need to use exercise and physical activity to stimulate the brain’s ancient mechanism of concentration (**Figures 5**, **6**).

Despite the obvious benefits of exercise, our modern lifestyle has led adults and young people alike to reduce their physical activity, and a major culprit is digital devices such as smartphones (100). While several strategies have emerged to mitigate or even prevent smartphone addiction, such as controlling smartphone use, enhancing self-control and exercising, future research should continue to explore risk factors for smartphone addiction as well as effective prevention measures and treatments. Of course, when controlling smartphone use, smartphone addicts may experience some physical and psychological discomforts. Smartphone addicts who suddenly reduce their phone use may experience withdrawal - like symptoms, including restlessness, anxiety, and a sense of being at a loss. This is because the brain has become accustomed to the stimulation and rewards from smartphone use, and the sudden change disrupts the neural pathways and chemical balances related to addiction (76).
